# Editorial: Language, corpora, and technology in applied linguistics

**DOI:** 10.3389/fpsyg.2023.1325925

**Published:** 2023-11-06

**Authors:** Swaleha Bano Naqvi, Muhammad Afzaal, Geng Qiang

**Affiliations:** ^1^International Business & Marketing Department, National University of Sciences and Technology (NUST), Islamabad, Pakistan; ^2^Institute of Corpus Studies and Applications, Shanghai International Studies University, Shanghai, China

**Keywords:** language, translation, applied linguistics, corpora, technology

The aim of this Research Topic was to stimulate thinking and research on emergent and important topics across the intersecting domains of applied linguistics, corpus-linguistics, translation and technology and to serve as a showcase for inventive cross-disciplinary deployment of tools and frameworks. This editorial indexes the rich diversity of the contributions made to *Language, corpora, and technology in applied linguistics* which not only extend knowledge in the target disciplines but also offer applications for the practice contexts of researchers, translators and teachers.

In extension of the focus on applied linguistics, a number of featured studies furnish pedagogical insights with the potential to improve instruction and the learning outcomes of learners across academic levels as well as of translation trainees. From the perspective of instruction, researchers offered useful insights from linguistic investigations with applications for the classroom. For instance, in a study based on meta-analysis of Chinese and global studies on the effect of metaphorical instruction on learners' outcomes, Zhou et al. identified that when teaching features prolonged instruction in metaphors which is integrated with practice, L2 learners are likely to make gains in achieving metaphorical competence. Similarly, an intervention-based study by Alawerdy and Alalwi used analysis of empirical data from first year Saudi EFL learners at university to showcase the impact of explicit instruction in the use of cohesive devices within essays, offering insights into how the writers' ability to write cohesively can be enhanced. Using comprehension and performance tasks with Arabic speaking children at a Riyadh international school, Alothman and Alsager investigated their acquisition of L2 spatial deixes. Observing the absence of proximity bias as an anticipated obstacle, the researchers found that the children's comprehension of spatial deixis in English was better than their production of the latter, suggesting the need for teachers take this into account when designing communicative activities. With implications for self-directed learning, an insightful study by Lyu and Han shows how a data driven approach (making use of a corpus) to bring about improvements helped a Chinese medical in her self-translations of a medical abstract. This approach indicates how other learners can adopt similar strategies (e.g., use of English equivalents for Chinse terms, keywords to identify collocations and utilization of accompanying words to discover contexts) to improve their L2 writing. With classroom interactions serving as a lever to facilitate better learning, a study by Wei et al. explored how interpersonal meanings between teachers and students are realized through the use of tag questions in British university seminars in BASE corpus. Wei et al. found that most tag questions are initiated by the teachers and serve to capture the attention of the listeners rather to seek information and that they can also serve as a means for teacher to retain control of the activities in the session, although the limited ways in which the tag questions are used within the context of class interactions suggest the need to pay greater attention to realizing the full potential of tag questions. In language learning contexts, paying attention to the influence of linguistic background of the learners can help to provide better instruction. In this vein, Song's corpus-based investigation of the mood functions of summing up adverbs found that the pragmatic mechanism of “violation” helped to realize similar functions of the “Hezhe” (合着), “ganqing” (敢情), and “nao le bantian” (闹了半天). These findings suggest the need to pay attention to words with provenance in Chinese dialects, particularly at midway and advanced levels.

Factors such as familiarity, meaning, transparency or decomposability in the context of figurative phrases tend to be considered autonomous. In this context, Alkhammash(a) analyzed data from participants belonging to a variety of language groups to show that the ability of the speakers to guess the meaning of a phrase correctly increases scores in transparency and decomposability, thus flagging the need for closer attention to these factors when designing instruction. Within the context of translation research, Man et al. propose the development of a longitudinal learner translation corpus that can facilitate assessment feedback on translation proficiency with the help of an ecological approach that takes into account spatial and temporal contexts for the texts.

Other contributions demonstrated a more specific linguistic focus, albeit demonstrating variation in the topics, themes and methodologies used to develop the insights. With implications for research on syntactic complexity (a gauge for L2 writer proficiency), a corpus analysis of syntactic complexity evident in the essays of Japanese university EFL writers (NICE corpus), Lyu et al. showed substantial statistical difference in the writings of the L2 writers and those of native writers at the same academic level, thus suggesting the need for researchers to pay closer attention to this factor when taking into account the evolution of L2 writer competence. Cui and Kim explored structural and functional bundle lengths in a self-compiled corpus of student dissertations to show considerable variation in bundle categories (research-oriented, text-oriented and participant-oriented) across 3-, 4-, and 5-word bundles. Stance expression is another area of challenge for L2 writers. Using a corpus-based approach to compare Chinese and American students' rhetorical moves and stance features in 112 dissertation abstracts, Liu et al. found that abstracts by Chinese academic writers tended to focus on presenting their research rather than situating or discussing it. Additionally, stance expressions across the abstracts written by both groups of students demonstrated variation, with Chinese students demonstrating a preference for modal verbs in addition to stance verbs + that clauses to express their position. Serving as an innovative contribution, a study by Wang Q. adopted a cognitive semantic approach integrated with interview data collected from disciplinary researchers to provide insights into how the geo-academic location of academic writers can predict their use of interest markers (e.g., interesting, intriguing, fascinating) to indicate their authorial stance. Based on this finding, enabling academic writers to notice these choices may help them to adapt to the writing conventions of the target and dominant scholarly community. From a semiotic perspective, Alharbi and Mahzari investigated the pragmatic functions of emojis within a dataset of Arabic tweets, revealing that emojis reflecting sorrow, love, tiredness among others were used prevalently, whereas the functions ranged from reaction, action, modification of tone to softening. These insights can allow for cross-cultural comparison of variations in how semiotic resources are appropriated and used. Exploring corpora comprising US, Chinese and UK media programs, Fu and Ho found that discourse markers such as *so, and, but* tend to be used the most in American programming which finding has implications for the realization of more effective bilateral communication between media program hosts and guests. Al-Mutairi and Mahzari examined a dataset of messages from computer-mediated communication related to advice-seeking by patients and advice-giving by doctors in the Arab medical context. The study found while the patients tended to ask yes/no questions and elaborate on their medical problems, the doctors made use of discoursal strategy dependent on offering clarification/information and direct advice.

A number of research contributions showcased the use of technology-integrated linguistic tools to investigate political, diplomatic and academic discourses. For example, undertaking a corpus-based analysis of political speeches of US presidents, Xu shows how different categories of pragmatic evidentiality in diplomatic discourse helps the orators to demonstrate distance or proximity to their propositions as well as to express their stances toward the topics of their speeches. Investigating the shifts in Chinese diplomatic discourse over the period of 1949–2018 by applying corpus-based discourse analysis, Tian and Li find that the evolution of China's engagement with countries in the world, its cooperation efforts and stride toward international sustainable development are represented in tandem within its discourses. These findings index the expansive potential of corpora in helping to probe and unpack linguistic data across spoken and textual data. Exploring academic discourses, Hu used a discourse-based approach to examine evolutions in American academia's cognitive construction of Confucius institutes (aimed at achieving Sino educational and cultural cooperation across the globe). Hu shows how positive reception in the early years (2004–2013) was succeeded by a cognitive transformation of the institutes as a political agency and swift increase in the closure of the institutes (2014–2017). Zhang, Zhao et al. analyzed the multiple sections mediating the presentation of language policies within a dataset of constitutional texts, revealing that these tended to be discussed the most in the section dilating upon the fundamental rights of the citizen and that the geographical location wherein these texts originated also influenced the distribution of the policies. Using Biber's multi-dimensional (MD) analysis as an analytical lens, Zhang, Afzaal et al. investigated Sino and American diplomatic discourses, revealing that while Chinese discourses are packed with information and independent of context, American discourses are not only context-dependent but also emotive as well as interactional.

The breadth of translation research is evidenced in the diversity of the contributions related to this thematic strand within the Research Topic. Some of the studies focused on the implications of translators' linguistic choices for their translated texts while others focused on the influence of culture, history and background on. For instance, the incidence of modal verbs which are used to express the speaker's attitudes was investigated within a corpus of Chinese-English government press conference interpretation data by Zhang and Cheung. Their study showed a greater instantiation of modal verbs in English translations when compared with the original Chinese texts, suggesting that the deployment of modal verbs expressive of attitudes in translated texts was due to the desire to achieve specific pragmatic functions. In another example, Meng and Pan apply corpus-analysis to English translation of Wang Anyi's novel *The Song of Everlasting Sorrow*. They find that translators make conscious stylistic choices when rendering source cultures texts into target culture translations to make these texts comprehensible to audiences accustomed to very different styles. Examining English translations of Mao Zedong's speeches with the help of corpus-based analysis, Huang and Shi reveal not only idiosyncratic language use but also the evident influence of source language transfer into the target language, expressed in the underuse of the person pronoun and preference for the modal verb *must* rather than *should*. Other studies focused on the translation context, taking into account the impact of historical background and cultural identity. For instance, investigating a self-constructed corpus of six English translations of the Chinese classic Daxue by Chinese and international translators, Wang H. shows that the translational choices made by them are influenced by their cultural identity as well as historical background and motivations to carry out the translations. Yang et al. show how the use of modal verbs in representative English translations of *Shih chi* is influenced not only by the professional identities of text translators but also by the historical as well as cultural contexts in which they lived and worked. In an innovative study making use of narrative inquiry, Geng chose to adopt a human-centered approach to explore the experience of five seasoned and senior translators as evident in their self-narratives. The study shows that translators are strongly influenced by their social, political and cultural settings while carrying out their work and that their narratives are structured chronologically with focus on critical incidents in their journey through life and work as translators.

Other studies adopted distinctive theoretical and methodological lens to explore language data, ranging from how poetic texts were adapted into the visual medium to how digital public platforms can reflect the asymmetries of a gender imbalanced society and world. For instance, systemic functional semiotics was used as a heuristic by Chen and Zhong to compare distribution of process types of language in comic adaptations of Chinese poetry. Their research reveals how poems are rendered into perceptions as well as actions and verbal processes within the comics to facilitate dramatization, development of the storyline as well as visualization of metaphors amongst other functions. Analyzing a self-constructed corpus of 7,000 tweets on @FacesofCovid with the help of social actor representation and corpus linguistics, Almaghlouth shows how gender asymmetry (reflected in the underrepresentation of female victims) persists even on a public mourning platform established to commemorate the COVID dead from around the world.

The editorial progresses toward its conclusion by focusing on three key studies based on a bibliometric and meta-analytic review of literature. Tackling a sensitive topic, Peng and Hu performed a bibliometric analysis of COVID-related research with the help of CiteSpace software. They found that pandemic research reflected a narrow topical focus as well as inadequate attention to discourse-based analyses. In another bibliometric study with implications for trends in metaphor studies within crisis communication, Alkhammash(b) shows how investigations in COVID metaphors tend to be underpinned by Critical Discourse Analysis and span conventional and emerging genres (e.g., social media and multi-modal data). From a more theoretical perspective, Han et al. undertake a novel review of research publications on Conceptual Metaphor Theory with the help of CiteSpace. They identified keywords indexing predominant themes in the publications, dominant research domains for present research as well as the need for more interdisciplinary research on CMT. Such studies serve an important function, identifying as they do dominant themes and topics as well as under-researched areas so that future researchers can turn their attention to urgent topics, avoid research in already saturated areas as well as pursue epistemic and methodological trajectories which can truly push the frontiers of knowledge in their domains.

Given that technology serves as a key thematic strand in the Research Topic, it is worth noting that many of the contributions make strategic use of technology. Featured studies integrating extended use of technology include the utilization of CiteSpace to enable keyword visualization and cluster analysis in bibliometric and metanalytic studies (Peng and Hu; Han et al.), #LancsBox corpus processing software to analyze tweets on @FacesofCOVID (Almaghlouth), Multidimensional Analysis Tagger (MAT) supported analysis of Sino and American political discourse (Zhang, Afzaal et al.) as well as meta-analysis statistical tool for determining whether metaphorical competence can be taught (Zhou et al.).

Summing up, this Research Topic evidences the exciting breadth and diversity of linguistic research across the disciplinary spectrum and global contexts. It also speaks to the potential of technology to be integrated into linguistic, corpus and translational research so as to allow for the leveraging of its power to tackle big data, thus enabling the production of meaningful results and findings applicable to wider populations as well as exploration of emergent topics, issues, and challenges.

## Author contributions

SN: Conceptualization, Writing—original draft, Writing—review & editing. MA: Writing—review & editing, Writing—original draft. GQ: Writing—review & editing.

